# Polypharmacology of Berberine Based on Multi-Target Binding Motifs

**DOI:** 10.3389/fphar.2018.00801

**Published:** 2018-07-24

**Authors:** Ming Chu, Xi Chen, Jing Wang, Likai Guo, Qianqian Wang, Zirui Gao, Jiarui Kang, Mingbo Zhang, Jinqiu Feng, Qi Guo, Binghua Li, Chengrui Zhang, Xueyuan Guo, Zhengyun Chu, Yuedan Wang

**Affiliations:** ^1^Department of Immunology, School of Basic Medical Sciences, Peking University Health Science Center, Beijing, China; ^2^Key Laboratory of Medical Immunology, Ministry of Health, Peking University, Beijing, China; ^3^State Key Laboratory of Natural and Biomimetic Drugs, School of Pharmaceutical Sciences, Peking University, Beijing, China; ^4^Department of Pathology, First Affiliated Hospital of Chinese PLA General Hospital, Beijing, China; ^5^Pharmacy Departments, Liaoning University of Traditional Chinese Medicine, Shenyang, China

**Keywords:** berberine, polypharmacology, multi-target binding motifs, drug-target space, Alzheimer’s disease

## Abstract

**Background:** Polypharmacology is emerging as the next paradigm in drug discovery. However, considerable challenges still exist for polypharmacology modeling. In this study, we developed a rational design to identify highly potential targets (HPTs) for polypharmacological drugs, such as berberine.

**Methods and Results:** All the proven co-crystal structures locate berberine in the active cavities of a redundancy of aromatic, aliphatic, and acidic residues. The side chains from residues provide hydrophobic and electronic interactions to aid in neutralization for the positive charge of berberine. Accordingly, we generated multi-target binding motifs (MBM) for berberine, and established a new mathematical model to identify HPTs based on MBM. Remarkably, the berberine MBM was embodied in 13 HPTs, including beta-secretase 1 (BACE1) and amyloid-β_1-42_ (Aβ_1-42_). Further study indicated that berberine acted as a high-affinity BACE1 inhibitor and prevented Aβ_1-42_ aggregation to delay the pathological process of Alzheimer’s disease.

**Conclusion:** Here, we proposed a MBM-based drug-target space model to analyze the underlying mechanism of multi-target drugs against polypharmacological profiles, and demonstrated the role of berberine in Alzheimer’s disease. This approach can be useful in derivation of rules, which will illuminate our understanding of drug action in diseases.

## Introduction

Recently, it has been appreciated that single drug acts on multiple rather than single targets, coined as polypharmacology, which opens a new avenue for drug discovery and development (Anighoro et al., 2014; Rastelli and Pinzi, 2015). Current studies in this emerging field focus on two major aspects: (i) polypharmacology across multiple disease-relevant targets can improve therapeutic efficacy (Monteleone et al., 2017); (ii) unintended drug-target interactions can lead to side effects (Peters, 2013). The versatile and promising scaffolds targeting protein–protein interactions offers opportunities for therapeutic intervention for the treatment of human diseases (Liu L.J. et al., 2017; Wang K. et al., 2017). Recent progress in protein labeling technology has enabled the development of probes for the determination of various biological molecules (Kang et al., 2017; Liu J.B. et al., 2017; Vellaisamy et al., 2018). Despite advances in these areas, considerable challenges still exist for polypharmacology modeling. The underlying assumption of the rational design will result from a detailed analysis of the nature of drug-target interactions for the proven effective and safe polypharmacological drugs, such as berberine (Wang W. et al., 2017).

Berberine is a natural isoquinoline alkaloid presented in various medicinal plants, including *Berberis vulgaris* (Barberry), *Coptis chinensis* (Coptis), and *Hydrastis canadensis* (Goldenseal) (Samanani et al., 2005). In traditional medicine, berberine has been widely used to treat diarrhea for a long history (Taylor and Greenough, 1989). Recently, berberine was reported to exhibit favorable therapeutic effects on infection (Chu et al., 2016), cancer (La et al., 2017), diabetes (Gong et al., 2017), obesity (Hvistendahl, 2012), hyperlipidemia (Kong et al., 2004), cardiovascular disease (Tan et al., 2016) and neurodegenerative disorders (Ahmed et al., 2015). The clinical efficacy of berberine is mediated by interacting with a mixture of biological pathways, which are involved in metabolism (Moghaddam et al., 2014; Zhang et al., 2014), transmission of signals (Chen et al., 2017; Spatuzza et al., 2014; Yue et al., 2017) and regulation of gene expression (Bhadra and Kumar, 2011). Increasing evidences indicate that berberine can act on a diverse range of molecular targets by binding to the active cavities that are to have certain structural and physiochemical properties, such as QacR (Schumacher et al., 2001), BmrR (Newberry et al., 2008), RamR (Yamasaki et al., 2013), phospholipase A_2_ (PLA_2_) (Chandra et al., 2012) and double helix DNA d(CGTACG)_2_ (Ferraroni et al., 2011). According to the co-crystal structures of these targets in complex with berberine, berberine binds highly site-specifically by interacting with residues flanking a hydrophobic groove in the pocket. Here, we analyzed and generated a MBM model for berberine to identify HPTs that tend to be nodes positioned in the ‘Goldilocks’ region of biological networks. These findings will provide new insights into the underlying mechanisms of berberine against polypharmacological profiles.

## Materials and Methods

### Computational Screening

All computational screening methods were performed using the Discovery Studio 2017R2 (DS; BIOVIA-Dassault Systèmes) running on Windows 8.1 operating system in a machine with an Intel^®^ Xeon^®^ E5-2699 v3 2.30 GHz octadeca core processor.

### Target Fishing of Potential Targets

The features of a pharmacophore model reflect the drug-target interaction mode. By fitting berberine against a panel of pharmacophore models, the potential targets for berberine were picked out by employing the Ligand Profiler protocol of DS, which is equipped with two available pharmacophore databases, i.e., PharmaDB and HypoDB. Literature retrieval was simultaneously carried out to refine and supplement the profiling results.

### Generation of Multi-Target Binding Motifs (MBM)

The three-dimensional crystal structures of the proven targets for berberine were retrieved from the Protein Data Bank^1^, including QacR (PDB ID: 1JUM), BmrR (PDB ID: 3D6Y), RamR (PDB ID: 3VW2), PLA_2_ (PDB ID: 2QVD), and double helix DNA d(CGTACG)_2_ (PDB ID: 3NP6). The structures were prepared and optimized using the Prepare Protein and Minimization protocols of DS (Chu et al., 2018). After optimization, the binding-site spheres were subsequently defined around the location of berberine. Based on the steric and electronic features in the binding sites of multiple targets with berberine, the MBM was generated using the Receptor-Ligand Pharmacophore Generation module in DS.

### MBM-Based Screening

The MBM-based screening starts with the analysis of potential targets by the DS package. The optimized berberine was docked into the refined potential targets using LigandFit. The LigandScore can define the binding sites, generate ligand conformations, dock each conformation, save the top docked structures, and apply scoring function to each docked structure for the best binding mode (Chu et al., 2016). The pharmacophore models were subsequently generated using the Receptor-Ligand Pharmacophore Generation module. The binding potential (BP) was calculated using the following equation: *BP* = *n*^α^ × *RMSD*^β^, where BP is also equal to the one-*K_D_*-minus-κ power of ten, *n* is the number of pharmacophore features matched with MBM, *RMSD* is the average distance in three-dimensional structure between pharmacophore and MBM. The values of α, β, and κ were determined as 2.599, -5.234, and -5.905, respectively, which was simulated from a series of proven *K_D_* values of berberine. The fit value is a combined measure of the similarity between pharmacophore and MBM, and computed by the following equation.

$$\text{Fit value=}\frac{1}{1+e^{-\log\left(BP/{BP}_{0}\right)}}$$

Here, *BP*_0_ was taken as the minimum *BP* of proven targets, i.e., BmrR with a *value* of 49.243. The fit value of BmrR (*Fit value* = 0.5) was used as a predetermined threshold to select HPTs of berberine from the potential pharmacophores.

### Molecular Dynamics Simulation

The selected poses of berberine with BACE1 (5ENM) and Aβ_1-42_ (2NAO) were subjected to 10 ns molecular dynamics (MD) simulations using DS. The stability of the complex was analyzed and confirmed by plotting root mean square deviation (RMSD). The RMSD is a measure of the deviation of the conformational stability of the proteins from backbone structure to the early starting structure and fundamental property investigation in MD. The binding energy of the stable complex was calculated using force field CHARMm which showed the sum of electrostatic and van de Waals interaction terms.

### SPR Analysis

The interaction of berberine (Sigma) with BACE1 (Sigma) and Aβ_1-42_ (Sigma) was analyzed by SPR spectroscopy with a Biacore T200 biosensor instrument (GE Healthcare) (Yamasaki et al., 2013). Each target was immobilized onto flow cells in a CM5 chip using an amine-coupling method. Binding analyses were carried out at 25°C and a flow rate of 30 μl min^-1^. Berberine in running buffer (1 × PBS, 3 mM DTT and 5% dimethyl sulfoxide, pH = 7.4) was run over each target at gradient concentrations as indicated. An empty flow cell, without any immobilized protein, was used as a reference. The binding curves were analyzed using the steady-state affinity analysis supplied with the BIA evaluation software (GE Healthcare).

### BACE1 Activity Assay

The inhibitory property of berberine on BACE1 was evaluated by a fluorescence resonance energy transfer (FRET) assay (Sigma) (Kennedy et al., 2016). Briefly, BACE1 enzyme and substrate were diluted in a Fluorescent Assay Buffer (Sigma) to produce 10× working solutions. Berberine was diluted into different concentrations in deionized water. This assay was performed in 96-well microplates using 100 μl, which comprised of 20 μl substrate solution, 2 μl BACE1 enzyme solution, and 5 μl berberine solution. The reaction was allowed to proceed for 2 h in the dark at 37°C. The product fluorescence before and after the reaction was measured by a POLARstar Omega multimode microplate spectrophotometer (BMG LABTECH) using 320 nm excitation and 405 nm emission wavelengths. The percentage of inhibition was calculated using the following equation: [1 - (*S* -*S*_0_)/(*C* -*C*_0_)] × 100, where *S* and *C* represent the fluorescence intensities in the presence and absence of berberine, respectively. Inhibition curves were created by graphing the percentage of inhibition versus the inhibitor concentration using linear regression.

### Aβ_1-42_ Aggregation Assay

Aβ_1-42_ was initially dissolved in HFIP (Sigma), evaporated, and re-dissolved in DMSO (Sigma) to a final concentration of 5 mM. To examine the effect of berberine on Aβ_1-42_ aggregation, peptide solutions containing Aβ_1-42_ monomers (10 μM) with and without berberine were incubated at 37°C for 48 h.

### Transmission Electron Microscopy (TEM)

The morphological changes of Aβ_1-42_ aggregation in the presence and absence of berberine were characterized by TEM using a JEM-1011 transmission microscope (JOEL, Tokyo, Japan). Each sample was spotted onto glow-discharged, formvar-coated 300 mesh copper grids (Ted Pella Inc.), incubated for 1 min, dried, and then negatively stained with 2% uranyl acetate (Chu et al., 2016).

### ThT Fluorescence Assay

The aggregation kinetics of Aβ_1-42_ were monitored by incorporation of ThT. ThT solution was diluted in phosphate buffer (10 mM phosphate, 150 mM NaCl, pH = 7.0) to a final concentration of 20 μM. The fluorescence intensity for 20 μM Aβ_1-42_ incubated with and without berberine was measured at 37°C using a POLARstar Omega multimode microplate spectrophotometer (BMG LABTECH) under kinetic fluorometric mode. Measurements were carried out at an excitation wavelength of 450 nm and an emission of 485 nm. To account for background fluorescence, ThT intensity from solution without Aβ_1-42_ was subtracted from solution containing Aβ_1-42_. Raw data were fitted using BMG LABTECH’s MARS Data Analysis Software.

### Animals and Treatment

Thirty 120-day male APP/PS1 transgenic mice were purchased from the Jackson Laboratory (Bar Harbor, ME, United States). All mice were housed at 25°C under a 12-h light-dark cycle and given free access to standard laboratory food and water. The mice were randomly assigned into three groups: control group (*n* = 10), 50 mg/kg berberine (*n* = 10) and 100 mg/kg berberine (*n* = 10). Berberine was administrated to these mice in their drinking water for 4 months, starting from 4 months of age just before they developed cognitive impairment and key pathologic features. The dose of berberine was chosen according to previous studies with no gender differences (Kong et al., 2004). All experiments were conducted in compliance with the Animal Care and Institutional Ethical Guidelines and approved by the Biomedical Ethical Committee of Peking University.

### Morris Water Maze Test

All mice at 8 months of age were subjected to the Morris water maze task for 5 days for evaluation on their learning and memory abilities (Vorhees and Williams, 2006). The apparatus consisted of a circular white metal pool (160 cm in diameter and 50 cm in height) filled with 26 cm deep water at constant temperature (22 ± 1°C) throughout the experiment. The water pool was divided into four quadrants by the water maze software and had a translucent acrylic platform (12 cm in diameter and 24 cm in height) placed in the center of the northwest quadrant and 1.0–2.0 cm below the water surface. The acquisition trial for the mice was the last training trials to find the hidden platform at a target quadrant. The escape latency was recorded by the finding-platform time. At day 5, the mice were subjected to the probe trial to access spatial memory. The time in the target quadrant and crossing the former platform area were recorded for testing spatial memory.

### Immunohistochemistry

Five-micrometer-thick sagittal paraffin sections of mouse brain were mounted on glass slides. The tissue sections were deparaffinized and rehydrated through graded ethanol washes. Antigen retrieval was conducted in 10 mM citrate buffer (pH = 6.0) and boiled for 2 min. The slides were incubated with 3% hydrogen peroxide for 10 min, and then blocked by 10% normal goat serum for 10 min at room temperature. After removing excess blocking buffer, indirect immunohistochemical staining was performed with anti-Aβ_1-42_ antibody (1:100, Covance Research Products, Berkeley, CA, United States) at 37°C for 1 h, washed thoroughly, and then incubated with biotinylated anti-mouse IgG antibody (1:100, Dako, Denmark) at 37°C for 45 min. The slides were detected using diaminobenzidine.

### Statistical Analysis

The results were conducted using Student’s *t*-test with SPSS 13.0 software (Chu et al., 2014). The data were expressed as means ± standard deviation (SD) of three independent experiments. Values of *p* < 0.05 were considered to be statistically significant.

## Results

### Generation of a MBM Model

**FIGURE 1:**
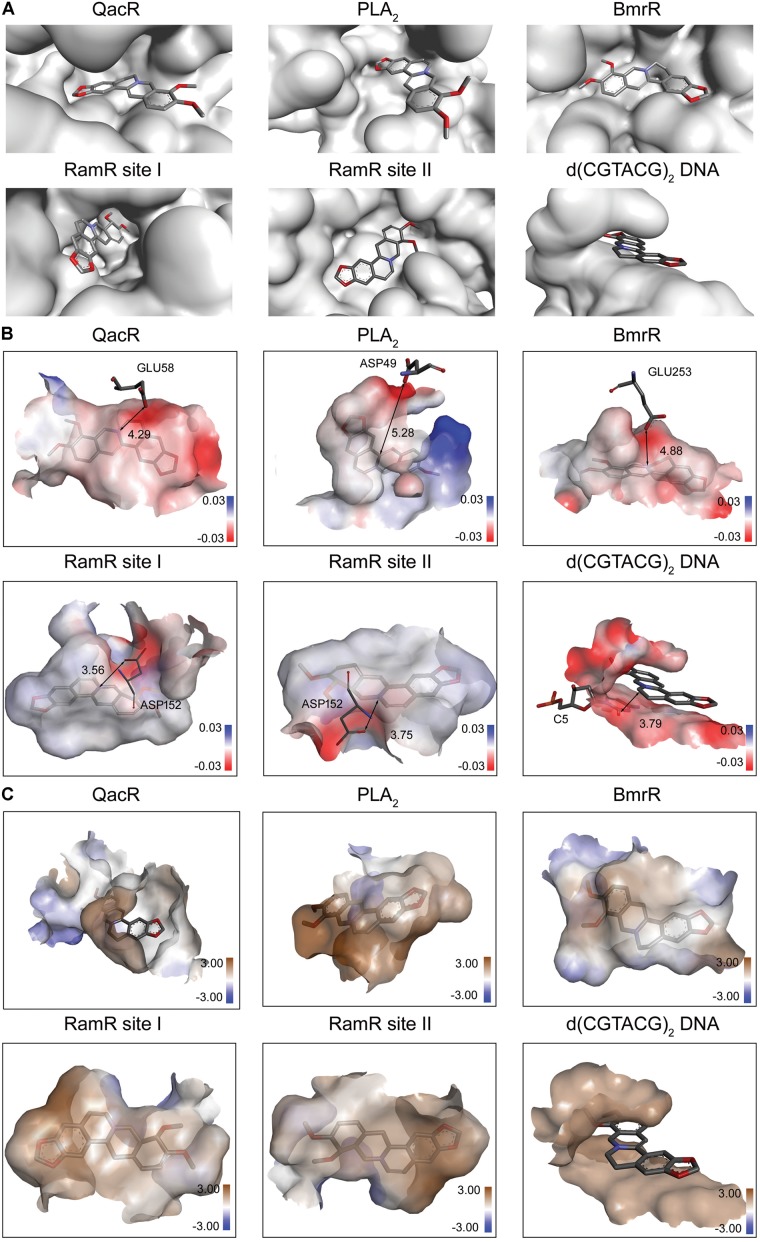
The physicochemical properties of multi-target binding pockets with berberine. Berberine is shown as sticks with carbon, oxygen and nitrogen colored gray, red and blue, respectively. **(A)** The substrate-binding sites of QacR, PLA_2_, BmrR, RamR, and d(CGTACG)_2_ DNA in complex with berberine. Two binding sites appeared in RamR, including site I and site II. **(B)** The chemical properties of multi-target binding pockets in complex with berberine. The surfaces are shown in gradient colors, where electropositivity is colored blue, and electronegativity is colored red. The key anions are labeled and depicted as sticks. **(C)** The physical properties of multi-target binding pockets in complex with berberine. The surfaces are shown in gradient colors, where hydrophobia is colored brown, and hydrophile is colored blue.

**FIGURE 2:**
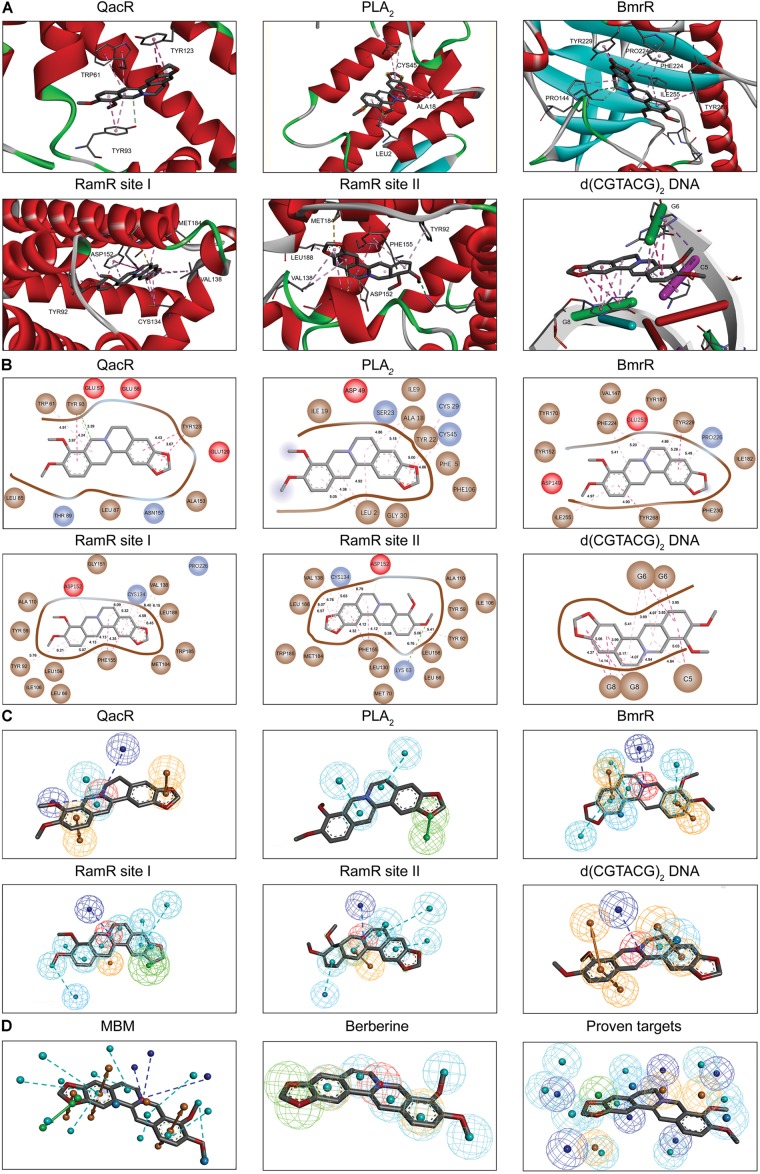
The MBM model of berberine. The berberine and key residues are shown as sticks with carbon, oxygen and nitrogen colored gray, red, and blue. Electrostatic interactions are shown as dashed lines with π–π, π-alkyl and hydrogen bonds colored purple, pink, and green, respectively. **(A)** The binding motifs of berberine within QacR, PLA_2_, BmrR, RamR, and d(CGTACG)_2_ DNA. Two binding sites appeared in RamR, including site I and site II. Secondary structural elements are depicted as ribbons (coils, α-helices; arrows, β-sheets). Color is based on secondary structures (α-helices, red; β-sheets, skyblue; loops, green). **(B)** The molecular interactions of berberine with surrounding residues. Hydrophobic, acidic and excluded residues are colored brown, red and blue, respectively. **(C)** The multi-target pharmacophore models of berberine. The colored spheres identify the position and the type of binding features (aromatic rings, orange; hydrophobic portions, cyan; cations, red; anions, blue; hydrogen bond acceptors, green). **(D)** MBM model obtained for berberine. For clarity, the binding features are represented as circles. Color-coding is given as indicated.

Berberine is a natural isoquinoline alkaloid drawing increased attention for its favorable therapeutic effects on various diseases. The clinical efficacy of berberine is determined by its activity across multiple targets, including QacR, PLA_2_, BmrR, RamR, and d(CGTACG)_2_ DNA. Since the co-crystal structures of these targets in complex with berberine have been reported in the PDB database, these targets are selected as proven targets to generate MBM model for berberine. Two binding sites obtained for berberine consist in RamR, including site I and site II (**Figure 1A**). The co-crystal structures locate berberine close to the negatively charged binding sites (**Figure 1B**). The distinguishing feature appears to be the presence of buried acidic residues, such as Glu, Asp, and nucleic acids, which provide electrostatic interactions with the positively charged nitrogen (N^+^) of berberine. Distances between the N^+^ of berberine and the key anions in QacR, PLA_2_, BmrR, RamR site I, RamR site II and d(CGTACG)_2_ DNA are 4.29, 5.28, 4.88, 3.56, 3.75, and 3.79 Å, respectively (**Figure 1B**). Berberine has a large hydrophobic surface, which is ideal for interacting with the aromatic and aliphatic residues, constituting the hydrophobic pockets (**Figure 1C**). The binding pose of berberine and surrounding residues within the multi-target binding pockets are nearly identical (**Figure 2A**). The side chains from residues provide π–π and π-alkyl interactions to aid in electrostatic neutralization for the positive charge of berberine (**Supplementary Figure S1**). Molecular interactions reveal that berberine can establish strong attractive charge contacts with the hydrophobic moieties of neighboring residues within 7 Å (**Figure 2B**). Accordingly, pharmacophores for multiple targets were generated based on the atomic details of the binding features (**Figure 2C**). Through essential pharmacophore superimposition, a predetermined MBM model of berberine was developed for further target screening (**Figure 2D**).

### MBM-Based Screening for HPTs

Multi-target binding motifs-based screening started with the choice of potential targets for berberine by target fishing with two available pharmacophore databases, i.e., PharmaDB and HypoDB. Literature retrieval was simultaneously carried out to refine and supplement the profiling results. A complete list of 86 potential targets involved with 38 diseases was summarized in the **Supplementary Table S1**. According to the MBM-based comparison function, a fit value equal to or greater than 0.50 was used as a heuristic threshold to select HPTs of berberine from the hit pharmacophores (**Table 1**). Among the 13 candidates, there are two HPTs associated with AD, including BACE1 and Aβ_1-42_, which indicated that berberine tended to be beneficial for the patients with AD. To validate the stability of berberine with BACE1 (5ENM) and Aβ_1-42_ (2NAO), we performed standardized MD stimulations through Pipeline Pilot using the CHARMm component in DS. As shown in the **Supplementary Figure S2**, the most plausible models of berberine with BACE1 and Aβ_1-42_ were stable.

**Table 1:** Highly potential targets of berberine.

Target name	Gene name	PDB ID	Fit value	Reference
TGF-β1 receptor	TGFBR1	3TZM	0.936114	Ogunjimi et al., 2012
Aβ_1-42_	APP	2NAO	0.906099	Walti et al., 2016
PksA	aflC	3HRR	0.818081	Crawford et al., 2009
BACE-1	BACE1	5ENM	0.788156	Wu et al., 2016
MEK-1	MAP2K1	3EQB	0.744816	Warmus et al., 2008
BRAF	BRAF	4E26	0.680164	Qin et al., 2012
CYP11A1	CYP11A1	3MZS	0.677786	Mast et al., 2011
CK2	CSNK2B	2OXD	0.636842	Battistutta et al., 2007
PDE10A	PDE10A	2O8H	0.596286	Chappie et al., 2007
MD-2	LY96	2E59	0.553963	Ohto et al., 2007
AMPK	PRKAA2	4ZHX	0.535163	Langendorf et al., 2016
LXR	NR1H2	3KFC	0.531021	Bernotas et al., 2010
HSP90	HSP90AA1	2XDU	0.503232	Murray et al., 2010


**Aβ_1-42_, amyloid-β_1-42_; AMPK, adenosine 5′-monophosphate-activated protein kinase; BACE-1, beta-secretase 1; cAMP, cyclic adenosine monophosphate; cGMP, cyclic guanosine monophosphate; CK2, casein kinase 2; COX-2, cyclooxygenase 2; CYP11A1, cytochrome P450 family 11 subfamily A member 1; Hsp90, heat shock protein 90; LXR, liver X receptor; MD-2, myeloid differentiation 2; MAPK, mitogen activated protein kinase; MEK-1, MAPK kinase 1; NF-κB, nuclear transcription factor kappa-light-chain-enhancer of activated B cells; PDE10A, phosphodiesterase 10A; PksA, polyketide synthase A; TGF-β1 receptor, transforming growth factor beta 1 receptor; Wnt, wingless-related integration site.**

### Identification of New Targets

The *Divine Farmer’s Classic of Materia Medica*, finished in the Han Dynasty (206 BC-220 AD), documented that “Chinese goldthread can prevent the loss of memory with extended treatment,” which is described as an “upper herb.” The major active principle, i.e., berberine, has recently been demonstrated as an effective therapeutic remedy to prevent or delay the pathogenesis of AD. However, the underlying mechanisms of berberine against AD are poorly understood.

**FIGURE 3:**
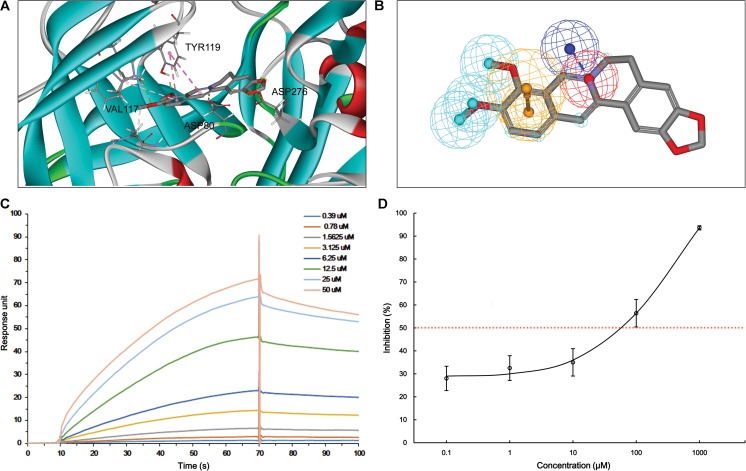
The action of berberine on BACE1. **(A)** The binding motif of berberine within BACE1. Secondary structural elements are depicted as ribbons (coils, α-helices; arrows, β-sheets). Color-coding is based on the secondary structures (α-helices, red; β-sheets, skyblue; loops, green). The berberine and key residues are shown as sticks with carbon, oxygen and nitrogen colored gray, red, and blue. Electrostatic interactions are shown as dashed lines with π–π and π-alkyl colored purple and pink, respectively. **(B)** The pharmacophore model of berberine obtained for BACE1. The colored spheres identify the position and the type of binding features (aromatic rings, orange; hydrophobic portions, cyan; cations, red; anions, blue). **(C)** The binding of berberine to BACE1. The association and dissociation of berberine with immobilized BACE1 was analyzed using SPR. **(D)** The inhibition of BACE1 enzymatic activity by berberine in a concentration-dependent manner. The data are represented as the mean ± SD of five replicates.

According to MBM-based screening, BACE1 and Aβ_1-42_ were predicted as HPTs for berberine, which lead to the aberrant production and aggregation of amyloid plaques in Alzheimer’s brains. Through application of molecular modeling, berberine was docked into the electronegative binding pocket of BACE1 (**Supplementary Figure S3**). The N^+^ of berberine engaged in electrostatic interactions with the key anion (Asp^80^) of BACE1, and the phenyl groups created π–π stacking with the Tyr^119^ active site residue (**Figure 3A**). A pharmacophore model was subsequently generated based on the interactions between berberine and BACE1 (**Figure 3B**). By fitting the pharmacophore against MBM, BACE1 was picked out as a HPT for berberine with a value of 0.788156 (**Table 1**). The complex of BACE1 with compound 10 (5ENM) was calculated using force field CHARMm which showed the binding energy to be -110.2 kcal/mol (Wu et al., 2016). The docked complex of BACE1 with berberine was more structurally stable and energetically favorable than the compound 10, with the binding energy reaching to -123.6 kcal/mol. Furthermore, the binding affinity of berberine to BACE1 was tested by Surface Plasmon Resonance (SPR). The equilibrium dissociation constant (*K*_D_) was calculated from SPR data to be 1.261 μM (**Figure 3C**). Moreover, we demonstrated that berberine effectively inhibited the activity of BACE1 with an IC_50_ of 62.96 μM, thereby decreasing the generation of Aβ_1-42_ (**Figure 3D**). Thus, BACE1 was identified as a new target of berberine.

**FIGURE 4:**
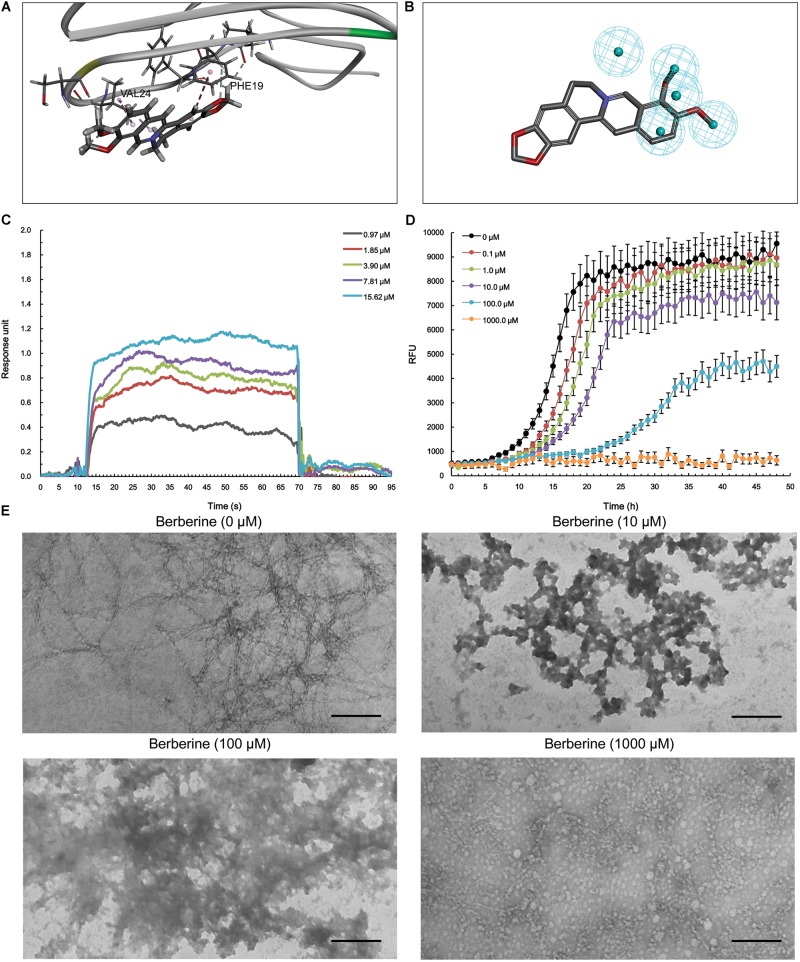
The action of berberine on Aβ_1-42_ oligomer. **(A)** The binding motif of berberine within Aβ_1-42_ oligomer. Secondary structures are depicted as ribbons (coils, α-helices; arrows, β-sheets). Color-coding is based on the secondary structures (α-helices, red; β-sheets, skyblue; loops, green). The berberine and key residues are shown as sticks with carbon, oxygen and nitrogen colored gray, red, and blue. Electrostatic interactions are shown as dashed lines with π-alkyl groups colored pink. **(B)** The pharmacophore model of berberine obtained for Aβ_1-42_ oligomer. The colored spheres identify the position and the type of binding features (hydrophobic portions, cyan). **(C)** The binding of berberine to Aβ_1-42_ oligomer. The association and dissociation of berberine with immobilized Aβ_1-42_ oligomer was analyzed using SPR. **(D)** The inhibition of Aβ_1-42_ seeding capacity by berberine. The aggregation kinetics of Aβ_1-42_ in the absence or presence of berberine were monitored by incorporation of ThT. Error bars represent the SD of eight replicates. **(E)** The TEM images of Aβ_1-42_ aggregation with or without berberine at 37°C for 48 h. The scale bars indicate 100 nm. Magnification: 50,000×.

With respect to Aβ_1-42_, berberine was docked into a large hydrophobic surface of the oligomer (**Supplementary Figure S4**). Molecular modeling revealed that berberine interacted with the residues by establishing hydrophobic bonds with Phe^19^ and Val^24^ (**Figure 4A**). The binding motif of berberine in Aβ_1-42_ was outlined from MBM-based screening with a fit value of 0.906099 (**Figure 4B** and **Table 1**). The docked complex of Aβ_1-42_ with berberine was calculated using force field CHARMm which showed the binding energy to be -73.6 kcal/mol. Moreover, the SPR responses indicated that berberine bound to Aβ_1-42_ oligomer with a *K*_D_ value of 1.491 μM (**Figure 4C**). The aggregation kinetics of Aβ_1-42_ in the absence or presence of berberine were monitored by incorporation of Thioflavin T (ThT). ThT fluorescence intensity showed that berberine was able to inhibit the seeding capacity of Aβ_1-42_ in a dose-dependent manner (**Figure 4D**). Furthermore, transmission electron microscope (TEM) was employed to observe Aβ_1-42_ aggregates. TEM image of Aβ_1-42_ alone appeared characteristic fibrils after 48 h incubation at 37°C (**Figure 4E**). The fibrillization was disturbed when co-incubated with increasing concentration of berberine, indicating that Aβ_1-42_ tended to be a new target of berberine (**Figure 4E**).

**FIGURE 5:**
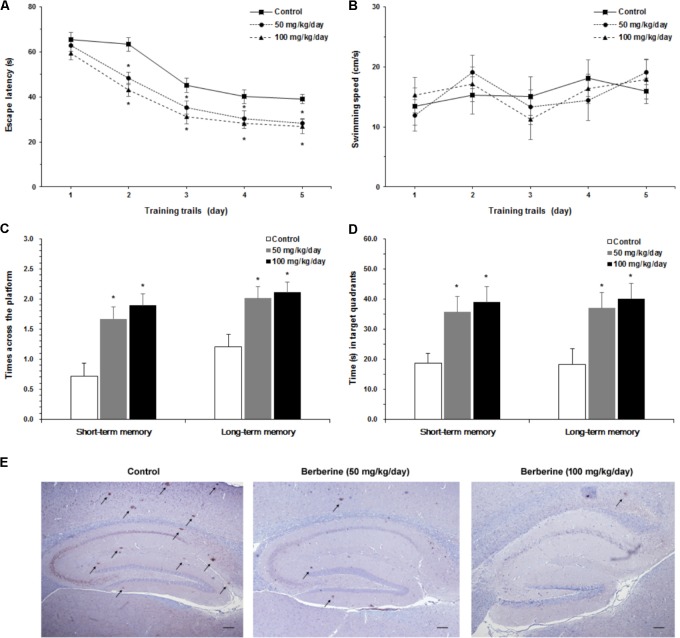
The neuroprotective properties of berberine in AD. Effect of berberine on spatial learning and memory of APP/PS1 mice were assessed by Morris water maze task. **(A)** The escape latency of mice were measured in 5-day training trials. Significant improvement in escape latency were observed in the berberine-treated mice (*p* < 0.05). **(B)** The swimming speed of mice during training trails. **(C)** The number of times crossing the target quadrant in both short-term memory and long-term memory. **(D)** The time of mice searching for the pre-placed platform in the target quadrant, in both short-term memory and long-term memory. Data shown are means ± SEM (*n* = 10). Significance was calculated by Student’s *t*-test. ^∗^*p* < 0.05. **(E)** Immunohistochemistry staining of the mice brain tissues with anti-Aβ_1-42_ antibody (4G8). Scale bar is 200 μm.

Furthermore, we studied the role of berberine in the prevention and treatment of AD. Spatial learning ability was observed on the basis of the time required to find the hidden platform. The berberine-treated APP/PS1 transgenic mice effectively improved their spatial learning ability in terms of daily escape latency through the 5-day consecutive training, compared with the control group (**Figure 5A**). However, no significant difference was assessed in swimming speed between the berberine-treated and the control groups (**Figure 5B**). Following the 5-day training, probe trials were conducted to assess both short-term (24 h) and long-term (72 h) memory tests on the 6th and 8th days, respectively. Significant differences were observed between the berberine-treated and the control mice (**Figure 5C**). In addition, the berberine-treated mice showed a significant improvement in the time spent searching for the pre-placed platform in both the short-term (*p* < 0.05) and long-term (*p* < 0.05) memory tests (**Figure 5D**). More importantly, the formation of amyloid plaque in the brain tissue was significantly inhibited for all the 50 mg/kg berberine (*n* = 10) and 100 mg/kg berberine (*n* = 10) treated mice, compared to the control group (*n* = 10) (**Figure 5E**). No significant difference was observed between 50 mg/kg berberine (*n* = 10) and 100 mg/kg berberine (*n* = 10) treated mice.

### MBM-Based Drug-Target Space

**FIGURE 6:**
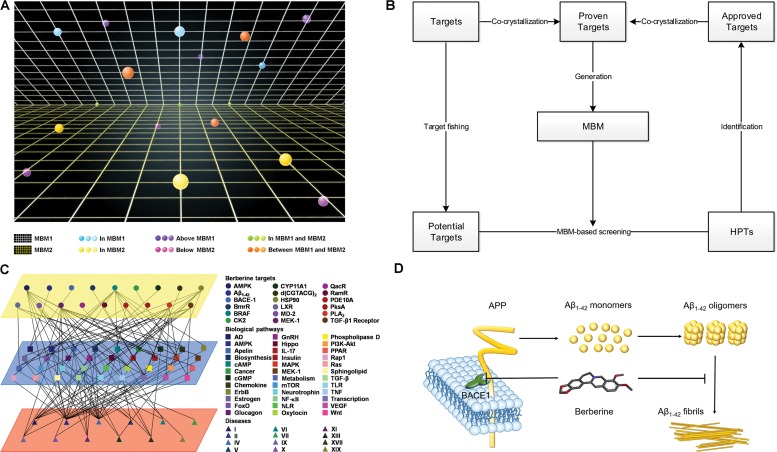
Multi-target binding motifs (MBM)-based network pharmacology. **(A)** Drug-target space. Two-dimensional plans and circles correspond to MBMs and targets, respectively. Color-coding is given according to the spatial relationship between drug-MBMs and targets. **(B)** The workflow of MBM-based screening. **(C)** Network pharmacology obtained for berberine. A network view of berberine is generated by mapping drug-target networks onto biological networks on the basis of KEGG pathway database. Circles, squares, and triangles represent targets, biological pathways and diseases, respectively. Diseases are classified according to the International Classification of Diseases 10th Revision. Color codes are given in the legend. **(D)** The underlying mechanisms of berberine against AD. Berberine prevents the aberrant production and aggregation of Aβ_1-42_ in the pathogenesis of AD.

Based on relationship between drugs and targets, we built a space model for drug-target network. In the drug-target space, MBM represents a two-dimensional plane across all targets for one drug, which reveals that (i) single drug against multiple targets; (ii) multiple drugs against single target (**Figure 6A**). The resulting interactomes will connect all drug targets into a highly interlinked space, with strong local clustering of targets for each drug based on MBM modeling. To study the space, we developed a novel approach on the basis of enormous information on drug-target interactions. The rational design strategy includes the choice of potential targets, the generation of a dedicated MBM model, the MBM-based screening of HPTs, and the identification of new targets (**Figure 6B**). The candidates lead continue in an iterative process of reentering MBM determination and reevaluation for further optimization (**Figure 6B**). Key principles in this emerging field were tested through integrating the network pharmacology for berberine based on MBM screening (**Figure 6C**). Overall, 13 HPTs were predicted, with 2 targets verified experimentally, including BACE1 and Aβ_1-42_ involved with AD (**Table 1**). Our results indicated that berberine was beneficial to decrease the generation of Aβ_1-42_ via inhibiting the enzymatic activity of BACE1, and disrupt the aggregation of Aβ_1-42_ into amyloid plaques (**Figure 6D**). Further studies on these HPTs will elucidate the underlying mechanism of berberine against polypharmacological profiles.

## Discussion

In drug discovery and development, the common analogy of drug action is that of a lock and key, with a correctly sized key (drug) fitting into the specific key hole (active cavity) of a lock (target). In recent years, it has been appreciated that a single drug can act as a ‘skeleton key’ that fits into multiple ‘locks’ of selective targets, coined as polypharmacology. Current studies in this emerging field focus on two major aspects, i.e., drug repurposing and multi-target drug design (March-Vila et al., 2017). The strategy relies on the knowledge of three-dimensional structures of biological targets, which is characterized as structure-based drug design (SBDD). During the past few decades, there has been a steep rise in the computer-aided drug design (CADD) that can assist in carrying out the process of SBDD from the selection of a target to the generation and evaluation of lead compounds effectively (Ramsay and Di Giovanni, 2017). Meanwhile, the explosion of structural information has provided exciting opportunities for SBDD (Réau et al., 2018). Herein we developed a novel approach for tackling the major sources of attrition in drug discovery, i.e., fact or fiction.

The strategy relies on the knowledge of drugs that bind highly site-specifically to the active cavity of targets. Prerequisite for an optimal supramolecular interaction is a perfect structural and physicochemical complementarity of drug and target. As a polypharmacological drug can act on multiple targets, the binding features of drugs are transformed into multi-target pharmacophores. The conformation that includes all the features is defined as a MBM model. The MBM theory explains how structurally diverse targets can bind with a common drug. According to the MBM, closely related targets identified through pharmacophore homology have the highest chance of cross reactivity and hence highest binding potential. Furthermore, the MBM model in turn can be used to design drugs that match the specific binding motif of a drug-target superfamily.

The inventive process of MBM-based screening starts with the choice of targets for a polypharmacological drug by target fishing with the pharmacophore databases. The hit targets are in need of literature evidence, and consequently classified into proven targets and potential targets. After analysis and curation of the binding motifs within the proven targets that have been co-crystallized with a common drug, a dedicated MBM model will be generated through essential pharmacophore superimposition. A MBM model is an ensemble of steric and electronic features that are necessary for recognition of a specific ligand by a broad and related category of molecules. A predetermined MBM model can be used to identify HPTs from the potential targets through pharmacophore comparison. Experiments are to be carried out to verify the affinity and efficacy. The approved targets will require co-crystallization with the drug to determine the binding modes. As increasing new targets become available, the MBM model will be updated to further refine it.

In this study, we gained insights into the network pharmacology for berberine by the application of MBM-based screening. Current studies have reported the co-crystal structures of five molecules in complex with berberine, including QacR, BmrR, RamR, PLA_2_ and d(CGTACG)_2_ DNA. Based on a detailed analysis of the interactions between berberine and its multiple proven targets, we generated a dedicated MBM model for berberine to identify HPTs from the 86 potential targets, including BACE1 and Aβ_1-42_. Each HPT is a node in a biological network that is linked to a particular disease, e.g., AD. AD is a progressive neurodegenerative disease, and the deposition of Aβ_1-42_ is the typical hallmark of AD pathology. In the amyloidogenic pathway, the amyloid precursor protein (APP) is sequentially cleaved by BACE1 to produce C-terminal fragment (C99), followed by cleavage by γ-secretase to produce Aβ peptides, including Aβ_1-42_ (Zhang et al., 2017). Recent studies have indicated that berberine could inhibit Aβ production (Cai et al., 2016). In particular, Zhang et al. (2017) indicated that berberine could reduce Aβ generation *via* decreasing the expression of BACE1. Herein, we focused mainly on the potential targets of berberine in AD. Consequently, we indicated that berberine could act as a high-affinity BACE1 inhibitor and prevented Aβ_1-42_ aggregation to delay the pathological process of AD, which further demonstrated that berberine tended to be beneficial for the patients with AD. These findings will provide new insights into the underlying mechanisms of berberine in the treatment of AD.

More importantly, a network view revealed that berberine can act on a node or combination of nodes whose perturbation results in a desired therapeutic outcome. Recent studies have identified several HPTs of berberine, including AMPK and MD-2, which confirmed the results of MBM-based screening (Chu et al., 2014; Zhang et al., 2017). Mapping the polypharmacology network for multi-target drugs onto the human disease network will enable a new network pharmacology approach to drug discovery. We believe that MBM-based network pharmacology will illuminate our understanding of drug action in diseases.

## Ethics Statement

This study was carried out in accordance with the recommendations of the Animal Care and Institutional Ethical Guidelines in China. The protocol was approved by the Biomedical Ethical Committee of Peking University.

## Author Contributions

MC designed the experiments. MC, XC, JW, LG, QW, ZG, and JF performed the experiments. MC, XC, LG, JK, MZ, QG, BL, CZ and XG analyzed the results. MC wrote the manuscript. ZC and YDW revised the manuscript. All authors read and approved the final manuscript.

## Conflict of Interest Statement

The authors declare that the research was conducted in the absence of any commercial or financial relationships that could be construed as a potential conflict of interest.

## Footnotes

1 www.rcsb.org

## Supplementary Material

FIGURE S1 Electrostatic interactions between berberine and key residues. The berberine and key residues are labeled and shown as sticks with carbon, oxygen, and nitrogen colored gray, red, and blue, respectively. The side chains from the residues provide π–π **(A)** and π-alkyl **(B)** interactions to aid in electrostatic neutralization for the positive charge of berberine. Electrostatic interactions are shown as dashed lines with π–π and π-alkyl colored purple and pink.

FIGURE S2 Molecular dynamics of berberine with BACE1 and Aβ_1-42_. The stability of berberine with BACE1 **(A)** and Aβ_1-42_ **(B)** was validated using a standardized MD protocol through Pipeline Pilot using the CHARMm component in DS.

FIGURE S3 The binding pocket of BACE1 with berberine. The binding surface is shown in gradient colors, where electropositivity is colored blue, and electronegativity is red. The berberine and Asp^80^ are shown as sticks with carbon, oxygen, and nitrogen colored gray, red, and blue, respectively. Distance between the N^+^ of berberine and Asp^80^ in BACE1 is 3.67 Å.

FIGURE S4 The binding pocket of Aβ_1-42_ oligomer in complex with berberine. The binding surface is shown in gradient colors, where hydrophobia is colored brown, and hydrophile is colored blue. The berberine are shown as sticks with carbon, oxygen, and nitrogen colored gray, red, and blue, respectively.

TABLE S1 Potential targets of berberine.
