# Supplementary figures and images for: Polypharmacology of Berberine Based on Multi-Target Binding Motifs
Source: Front Pharmacol. 2018 Jul 24;9:801. doi: 10.3389/fphar.2018.00801 (PMC6066535; doi:10.3389/fphar.2018.00801)

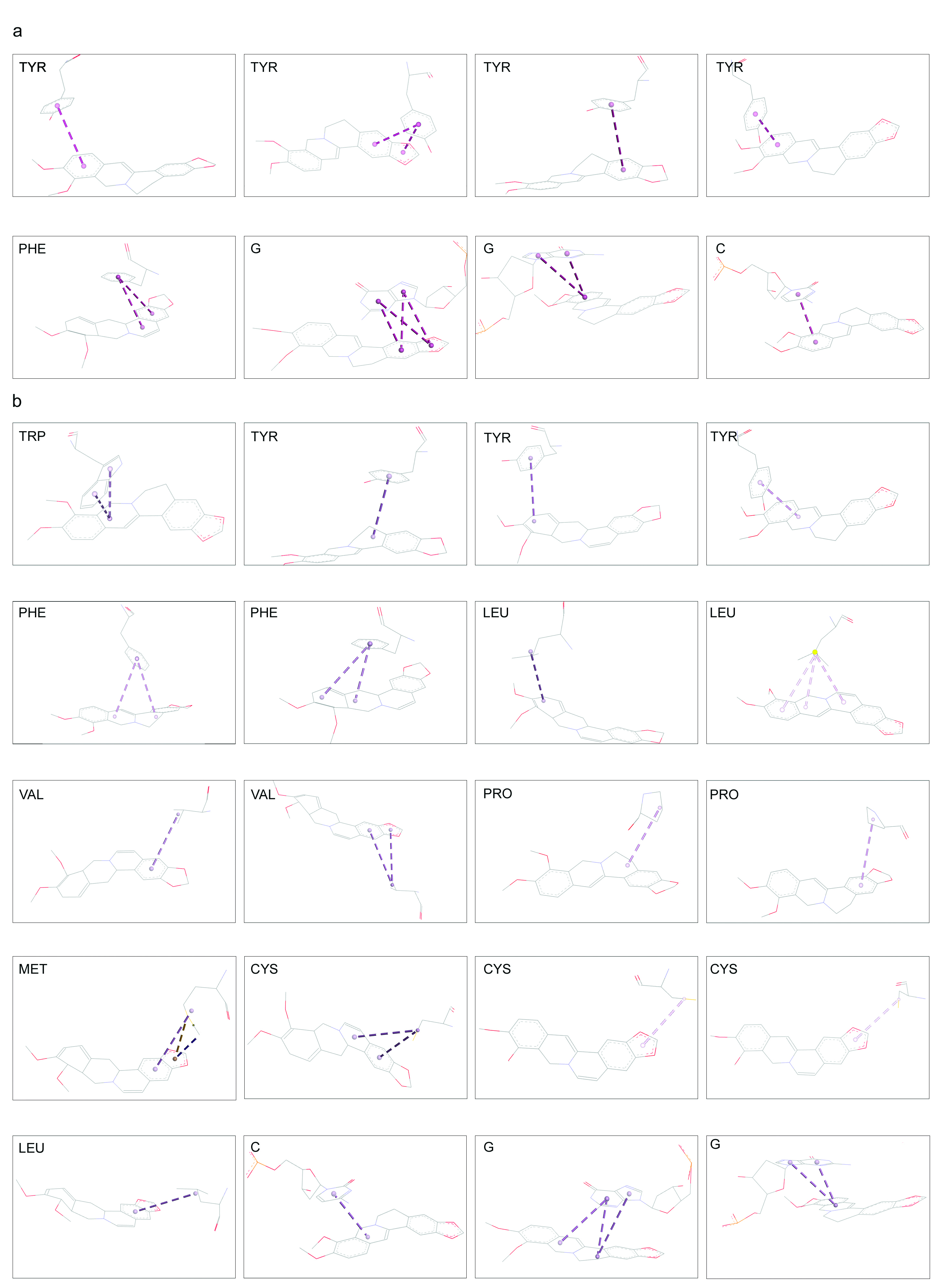

Supplement: FIGURE S1 — Electrostatic interactions between berberine and key residues. The berberine and key residues are labeled and shown as sticks with carbon, oxygen, and nitrogen colored gray, red, and blue, respectively. The side chains from the residues provide π–π (A) and π-alkyl (B) interactions to aid in electrostatic neutralization for the positive charge of berberine. Electrostatic interactions are shown as dashed lines with π–π and π-alkyl colored purple and pink. [file Image_1.JPEG]

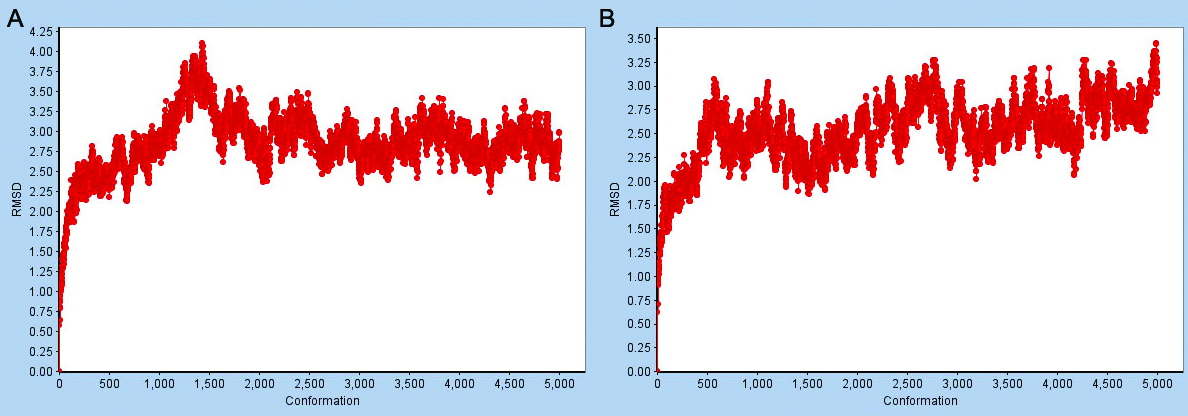

Supplement: FIGURE S2 — Molecular dynamics of berberine with BACE1 and Aβ1-42. The stability of berberine with BACE1 (A) and Aβ1-42 (B) was validated using a standardized MD protocol through Pipeline Pilot using the CHARMm component in DS. [file Image_2.JPEG]

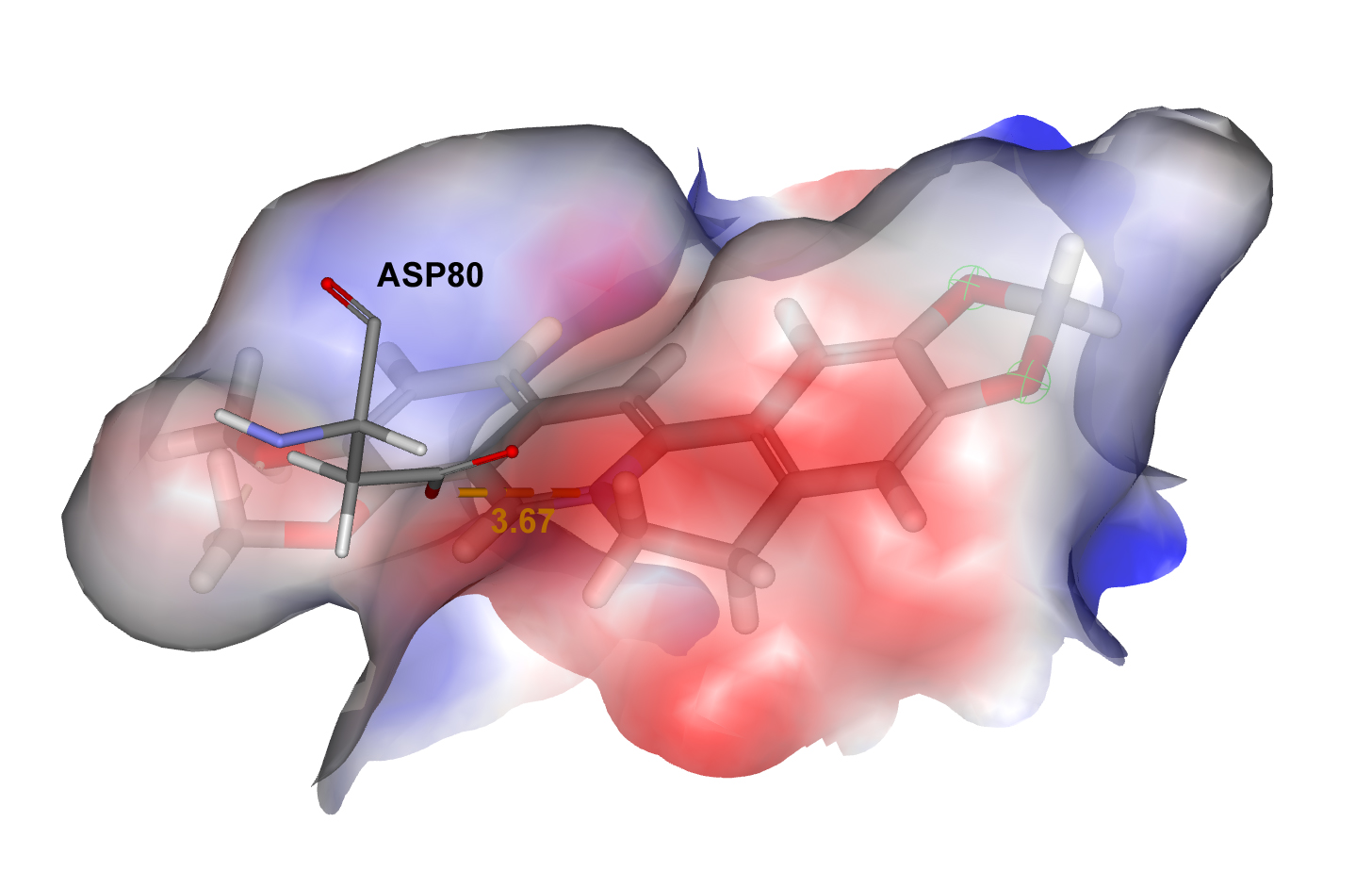

Supplement: FIGURE S3 — The binding pocket of BACE1 with berberine. The binding surface is shown in gradient colors, where electropositivity is colored blue, and electronegativity is red. The berberine and Asp80 are shown as sticks with carbon, oxygen, and nitrogen colored gray, red, and blue, respectively. Distance between the N+ of berberine and Asp80 in BACE1 is 3.67 Å. [file Image_3.JPEG]

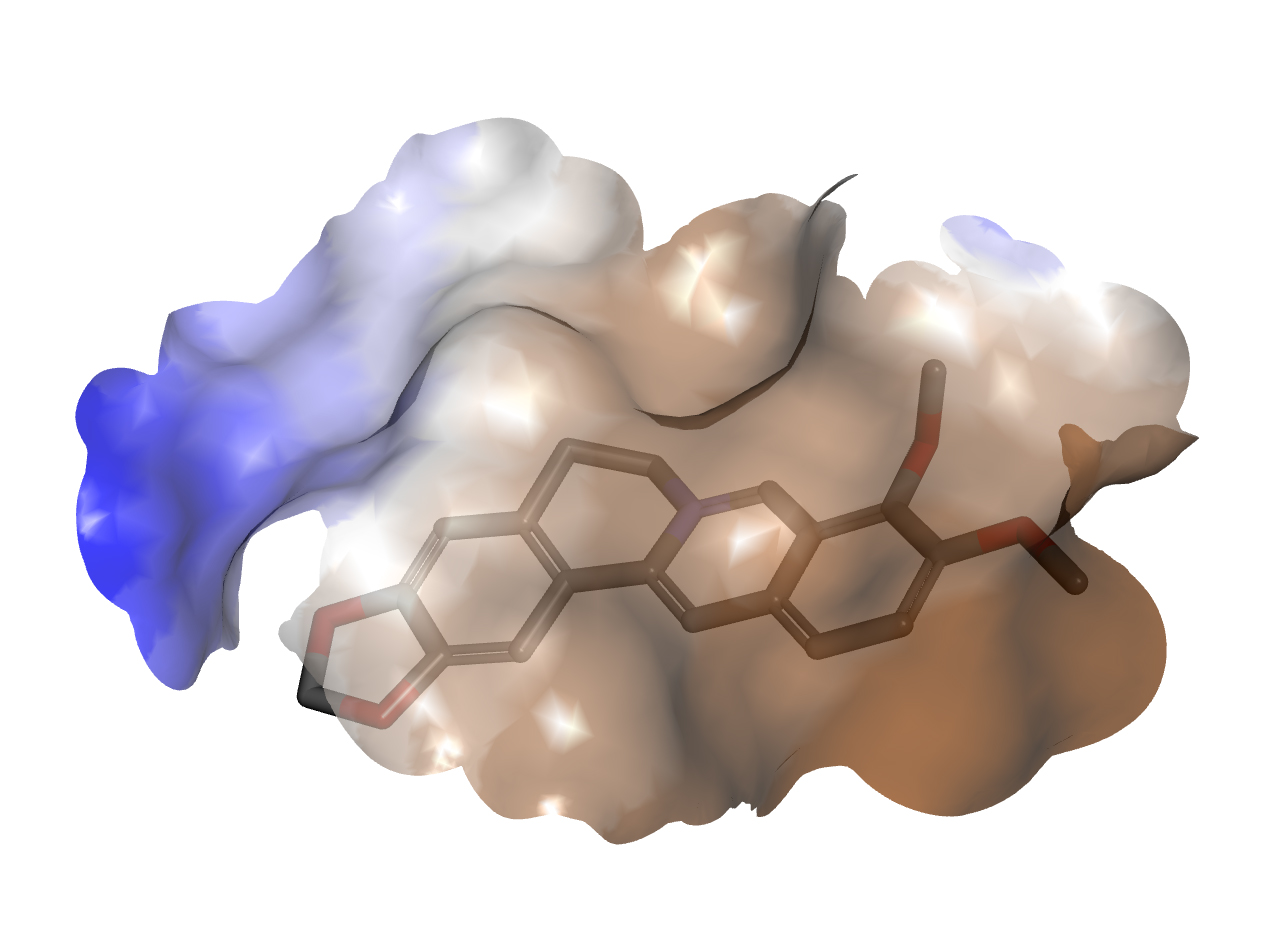

Supplement: FIGURE S4 — The binding pocket of Aβ1-42 oligomer in complex with berberine. The binding surface is shown in gradient colors, where hydrophobia is colored brown, and hydrophile is colored blue. The berberine are shown as sticks with carbon, oxygen, and nitrogen colored gray, red, and blue, respectively. [file Image_4.JPEG]
