# Supplementary material for: Polypharmacology of Berberine Based on Multi-Target Binding Motifs
Source: Front Pharmacol. 2018 Jul 24;9:801. doi: 10.3389/fphar.2018.00801 (PMC6066535; doi:10.3389/fphar.2018.00801)
Supplement: TABLE S1 — Potential targets of berberine. [file Table_1.DOC]

**Table S1. Potential targets of berberine**

| **ICD-10** | **Diseases** | **Potential targets** |
| --- | --- | --- |
| I | Diarrhea | Potassium channel |
|  | Infection | TLR-4, MD-2, PksA |
| II | Colon cancer | AMPK, Hsp90 |
|  | Gastric cancer | STAT-3, Survivin, Hsp90 |
|  | Lung cancer | Caspase-3, COX-2, CYP11A1, HIF-1α, Hsp90, MEK-1, PI3K, BRAF, VEGFR2 |
| IV | Aging | mTOR |
|  | Diabetes mellitus | AMPK, DLAT, DOT1L, MAPK4, LXR, NF-κB, Nrf2, PPAR, RHOA, SETD2 |
|  | Diabetic nephropathy | AR, NF-κB, Smad2, Smad3, SOD, SphK1, TGF-β1 receptor |
|  | Metabolic syndrome | ATF-2, MMP-2, p38 MAPK |
|  | Obesity | CD14, TLR-4 |
| V | Cocaine dependence | Tyrosine hydroxylase |
|  | Cognitive impairment | Bcl-2, Beclin-1, Cathepsin-D, hVps34, LC3-II, p62, P-gp |
|  | Depression | OCT-2, OCT-3 |
| VI | Alzheimer’s disease | Aβ_1-42_, BACE-1 |
|  | Multiple sclerosis | MMP-9 |
|  | Parkinsonism | AChE, BChE, Calyculin A, MAO-A, MAO-B, PDE10A, Tau |
| VII | Retinal degeneration | Photoreceptor, Rho |
| IX | Atherosclerosis | ABCA1, AMPK, AP-1, MMP-2, MMP-9, NADPH oxidase, p38 MAPK |
|  | Arrhythmic | hHCN4 channels |
|  | Myocardial ischemia | AMPK, Bax, Caspase-3, Cyto-c, HMGB-1, PI3K, TLR-4 |
|  | Hypertension | Myd88, NF-κB, RAS, TLR-4, TRPV4 |
|  | Acute myocardial injury | CAT, COX-2, HO-1, iNOS, LDH, SOD |
|  | Cardiac failure | Phospholamban |
|  | Cerebral ischemia diseases | Nrf2, PI3K |
|  | Ischemia-reperfusion injury | PI3K |
| X | Allergic rhinitis | p38 MAPK |
|  | Asthma | NF-κB |
| XI | Hepatic fibrosis | ALT, AMPK, a-SMA, AST, CYP2E1, HIF-1α, TGF-β1 receptor |
|  | Liver disease | AMPK, CYP2E1, HIF-1α, Nox-4 |
|  | Hepatic steatosis | Aβ_1-42_, AChE, APP, IDE |
|  | Irritable bowel syndrome | 5-HT |
|  | Reflux esophagitis | HO-1, NF-κB, SOD |
|  | Ulcerative colitis | Nrf2 |
| XIII | Osteoarthritis | AMPK, NF-κB, MMP-1, MMP-3, MMP-13, p38 MAPK |
| XIV | Ischemic acute renal failure | GSH, SOD |
|  | Kidney damage | 3-NT, 4-HNE, COX-2, CYP2E1, HO-1, iNOS, NF-κB, TNF-α |
| XIX | Hepatic injury | CYP2E1, SIRT1 |
|  | Traumatic brain injury | MMP-9 |

3-NT, 3-nitrotyrosine; 4-HNE, 4-hydroxynonenal; 5-HT, 5-hydroxytryptamine; Aβ_1-42_, amyloid-β_1-42_; ABCA1, ATP-binding cassette transport protein A1; AChE, acetylcholinesterase; ALT, alanine aminotransferase; AMPK, adenosine 5’-monophosphate-activated protein kinase; AP-1, activator protein 1; APP, amyloid precursor protein; AR, aldose reductase; a-SMA, alpha-smooth muscle actin; AST, serum aspartate aminotransferase; ATF-2, activating transcription factor 2; BACE-1, β-site amyloid precursor protein cleaving enzyme-1; Bax, Bcl-2 associated X protein; BChE, butyrylcholinesterase; Bcl-2, B-cell lymphoma 2; CAT, catalase; Caspase-3, cysteine-aspartic proteases 3; CD14, cluster of differentiation 14; CK2, casein kinase 2; COX-2, cyclooxygenase-2; CYP11A1, cytochrome P450 family 11 subfamily A member 1; CYP2E1, cytochrome P450 family 2 subfamily E member 1; Cyto-c, cytochrome-c; DLAT, dihydrolipoamide S-acetyltransferase; DOT1L, disruptor of telomeric silencing-1-like histone methyltransferase; GSH, glutathione; hHCN4, human hyperpolarization-activated cyclic nucleotide-gated 4; HIF-1α, hypoxia-inducible factor 1α; HMGB-1, high-mobility group box 1; HO-1, heme oxygenase-1; Hsp90, heat shock protein 90; hVps34, class Ш PI3K; ICD-10, International Classification of Diseases 10^th^ Revision; IDE, insulin-degrading enzyme; iNOS, inducible nitric oxide synthase; LC3-II, microtubule-associated protein 1 light chain 3-II; LDH, lactate dehydrogenase; LXR, liver X receptors; MAO, monoamine oxidase; MAPK4, mitogen activated protein kinase 4; MD-2, myeloid differentiation 2; MEK-1, MAPK kinase 1; MMP-2, matrix metalloproteinase 2; MMP-3, matrix metalloproteinase 3; MMP-9, matrix metalloproteinase 9; MMP-13, matrix metalloproteinase 13; mTOR, mechanistic target of rapamycin; Myd88, myeloid differentiation primary response gene 88; NADPH, nicotinamide adenine dinucleotide phosphate (reduced form); NF-κB, nuclear transcription factor kappa-light-chain-enhancer of activated B cells; Nox-4, nicotinamide adenine dinucleotide phosphate-oxidase 4; Nrf2, nuclear factor erythroid 2 p45-related factor 2; OCT, organic cation transporter; PDE10A, phosphodiesterase 10A; PI3K, phosphatidyl inositol 3-kinase; P-gp, P-glycoprotein; PksA, polyketide synthase A; PPAR, hepatic peroxisome proliferator-activated receptors; RAS, renin-angiotensin system; Rho, rhodopsin; RHOA, Ras homolog gene family, member A; SETD2, SET domain-containing 2; SOD, superoxide dismutase; SIRT1, sirtuin1; SphK1, sphingosine kinase 1; STAT-3, signal transducer and activator of transcription 3; TLR4, toll-like receptor 4; TGF-β1 receptor, transforming growth factor-β1 receptor; TNF-α, tumor necrosis factor-alpha; TRPV4, transient receptor potential vanilloid 4; VEGFR2, vascular endothelial growth factor receptor 2
